# Understanding Cancer Cachexia and Its Implications in Upper Gastrointestinal Cancers

**DOI:** 10.1007/s11864-022-01028-1

**Published:** 2022-10-21

**Authors:** Leo R. Brown, Barry J. A. Laird, Stephen J. Wigmore, Richard J. E. Skipworth

**Affiliations:** 1 Clinical Surgery, University of Edinburgh, Royal Infirmary of Edinburgh, Edinburgh, Scotland EH16 4SA UK; 2 Institute of Genetics and Cancer, University of Edinburgh, Western General Hospital, Edinburgh, Scotland EH4 2XU UK; 3 St Columba’s Hospice, Edinburgh, Scotland EH5 3RW UK

**Keywords:** Oesophageal cancer, Gastric cancer, Oesophagogastric cancer, Upper gastrointestinal cancer, Cachexia, Catabolism, Muscle-wasting, Body composition, Weight loss

## Abstract

Considerable advances in the investigation and management of oesophagogastric cancer have occurred over the last few decades. While the historically dismal prognosis associated with these diseases has improved, outcomes remain very poor. Cancer cachexia is an often neglected, yet critical, factor for this patient group. There is a persuasive argument that a lack of assessment and treatment of cachexia has limited progress in oesophagogastric cancer care. In the curative setting, the stage of the host (based on factors such as body composition, function, and inflammatory status), alongside tumour stage, has the potential to influence treatment efficacy. Phenotypical features of cachexia may decrease the survival benefit of (peri-operative) chemoradiotherapy, immunotherapy, or surgical resection in patients with potentially curative malignancy. Most patients with oesophagogastric cancer unfortunately present with disease which is not amenable, or is unlikely to respond, to these treatments. In the palliative setting, host factors can similarly impair results from systemic anti-cancer therapies, cause adverse symptoms, and reduce quality of life. To optimise treatment pathways and enhance patient outcomes, we must utilise this information during clinical decision-making. As our understanding of the genesis of cancer cachexia improves and more therapeutic options, ranging from basic (e.g. exercise and nutrition) to targeted (e.g. anti-IL1 α and anti-GDF-15), become available, there can be grounds for optimism. Cachexia can change from a hitherto neglected condition to an integral part of the oesophagogastric cancer treatment pathway.

## Introduction

Over 1.5 million new cases of gastric and oesophageal cancer are diagnosed globally each year [1]. Although decreasing overall, the incidence of gastric cancer varies markedly across the world. Differing diets and variation in practice regarding the investigation and treatment of *Helicobacter pylori* infection are two key factors that may account for this disparity. These, and other behavioural risk factors, make it one of the most preventable major cancers worldwide. Similarly, notable differences have been observed in the geographic distribution of oesophageal cancer. While squamous cell carcinoma remains the most common histological subtype worldwide, rates of oesophageal adenocarcinoma are rising and it has now transitioned to become the predominant subtype in North America, Oceania and much of Europe [2].

During the last 20 years, there have been significant developments in the management of patients with oesophagogastric (OG) cancer. The use of neoadjuvant therapies for patients with locally advanced disease [3, 4] alongside ongoing development and standardisation of surgical techniques [5–7] have contributed towards improved outcomes in those treated with curative intent. Identification and surveillance of Barrett’s oesophagus, a histological precursor for oesophageal adenocarcinoma, allows earlier detection and treatment of dysplasia. In such cases, patients may be suitable for endoscopic resection or ablation, rather than surgery [8]. Both of these treatment options are associated with markedly lower morbidity and mortality [9]. Despite these major advances, long-term survival for patients with oesophageal or gastric cancer remains far below that of other major cancer sites [10]. Together they account for 13.5% of all cancer-related deaths [1]. The reason for these adverse outcomes is undoubtedly multi-factorial. When compared with other tumour locations, a greater proportion of patients with OG cancer present with locally advanced or metastatic disease. Furthermore, surgical resection is technically challenging and rates of postoperative mortality remain high [11, 12]. Less often considered are the negative contributions of tumour-host interactions and the high incidence of cancer cachexia.

Cachexia is a syndrome seen in the terminal course of many chronic diseases, such as cardiac or renal failure and chronic obstructive pulmonary disease. It is, however, most frequently associated with advanced malignancy. The 2011 consensus definition described cancer cachexia as a “multifactorial syndrome characterised by an ongoing loss of skeletal muscle mass (with or without loss of fat mass) that cannot be fully reversed by conventional nutritional support and leads to progressive functional impairment” [13]. It is an involuntary, often rapidly progressive, wasting process with devastating consequences. The presence of cachexia is not a binary factor, but instead a progressive spectrum of disease, potentially affecting patients with all stages of tumour to varying degrees. Initially, a “pre-cachectic” state may be observed with more subtle clinical features, but this will often progress to “cachexia” and “refractory cachexia” [13]. By this point, the process is thought to be irreversible or ethically inadvisable to address.

In OG cancer, cachexia is likely to be a critical factor across pre-surgical assessment, post-surgery recovery, and in those where treatment is with non-curative intent. It can influence the efficacy of systemic anti-cancer therapy (SACT), quality of life, and ultimately survival [14]. Herein, this narrative review shall address the epidemiology, pathophysiology, and treatment of cachexia, in the setting of oesophageal cancer.

## Epidemiology of cachexia

**Fig. 1:**
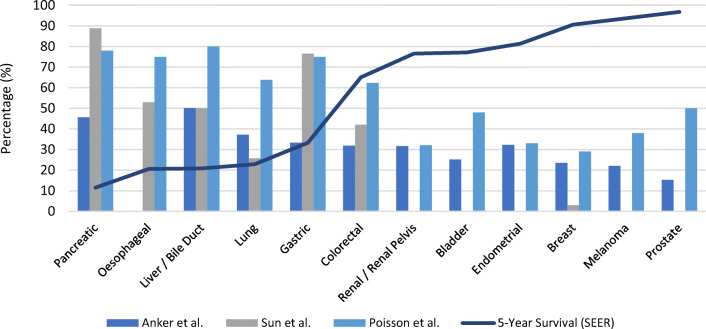
Estimated prevalence of cachexia vs. survival by tumour site. Adapted from data by Anker *et al.* [16]*,* Sun *et al.* [17]*,* Poisson *et al* [18] & the Surveillance, Epidemiology & End Results (SEER) program [19].

Several studies have attempted to quantify the prevalence of cachexia in patients with cancer. Preceding any published definitions, DeWys et al.’s 1980 paper explored weight loss across a range of cancer types in patients planned for chemotherapy, who were recruited to trials as part of the Eastern Cooperative Oncology Group [15]. This cohort revealed considerable variation in pre-treatment weight loss between sites. A modest 14% of patients with breast cancer had more than 5% weight loss in the preceding 6 months, while rates in lung (34–36%), pancreas (54%), and gastric cancers (62–67%) were far higher. Furthermore, weight loss was independently associated with poor survival. Other contemporary studies have assessed the variation in cachexia prevalence [16–18]. While heterogeneity in definitions and cohorts limit the accuracy with which results can be synthesised, higher rates of cachexia have consistently been noted in OG, hepatopancreatobiliary, and lung cancers. When compared with other cancers, such as colorectal, breast, or prostate, a greater proportion of patients with upper GI cancers have locally invasive or metastatic disease stage at presentation. As cachexia is more frequently seen in advanced staged malignancies, this is likely a contributing factor towards the observed high incidence. When plotted against survival rates for these cancer subtypes, an inverse relationship is evident (Fig. 1).

## The pathophysiology of wasting in oesophagogastric cancer

Throughout the world, malnutrition is all too frequently seen secondary to inadequate oral intake. Traditionally, cachexia was simply viewed as a similar nutritional issue; however, it has since become clear that the pathophysiology of disease-associated wasting is far more complex. Systemic inflammation drives malnutrition via hypermetabolism and neuro-endocrine dysfunction. Alongside inadequate oral intake and anorexia, there is elevated resting energy expenditure and increased catabolism of muscle and fat evident in cachectic patients. The resultant phenotypic changes, with depleted body stores and decreased levels of physical function, are often considered irreversible hallmark signs of impending poor clinical outcomes.

### Mechanisms of cancer cachexia

Systemic inflammation is a central tenet in the cancer cachexia process. Indeed, the Evans’ et al. alternative definition of cachexia [20] and the recent Global Leadership Initiative on Malnutrition (GLIM) consensus paper [21••] have highlighted the importance of inflammation through their diagnostic criteria. Evan’s et al.’s definition required significant weight loss with at least three of five phenotypic features, one of these being raised inflammatory markers (e.g. CRP or IL-6). In the GLIM criteria, at least one phenotypic criterion (weight loss / low body mass index (BMI) / reduced muscle mass) and one aetiological criterion (reduced food intake or assimilation/inflammation) are required to diagnose malnutrition. These aetiological criteria differentiate starvation, with protein-sparing metabolism, from disease-associated malnutrition where muscle loss is a hallmark feature. Again, the GLIM group recommended use of proxy measures of systemic inflammation such as C-reactive protein (CRP) or albumin. The exact pathophysiological basis for the chronic inflammatory response associated with cachexia is not entirely clear. It has been postulated that local tissue damage and tumour necrosis may stimulate systemic inflammation [22]. Pro-inflammatory factors released as part of the tumour secretome have also been identified [14]. There is evidence that many of these cytokines may even contribute towards the initiation, local invasion and metastasis of cancer [23].

### Immune regulation of cachexia

The question of the key mediator(s) for cancer cachexia has eluded researchers for decades. Many candidates have been implicated but results have frequently been conflicting, and interventional studies that have targeted individual factors have been often ineffective.

Interleukin-6 (IL-6) is likely to be an important marker in cancer cachexia syndrome [24]. Numerous studies, including in OG cancer cohorts [25, 26], have suggested an increased expression of IL-6 in cachectic patients when compared with weight-stable and healthy counterparts [27, 28]. IL-6 has also been correlated with reduced survival across a number of tumour sites and stages [29–31]. While a more limited number of studies have considered interleukin-8 (IL-8), higher levels have been similarly demonstrated in cachectic populations [27]. Considering pancreatic cancer, it has been shown to be the only cytokine upregulated with disease progression and positively correlated with weight loss [28]. Alongside IL-6, IL-8 has also been suggested to be the cytokine most closely linked to cancer cachexia in oesophagogastric cohorts [26].

Tumour necrosis factor alpha (TNF-α) has been shown to induce anorexia and have direct catabolic effects on both skeletal muscle and adipose tissue [32]. The majority of human cancer studies have found higher levels of TNF-α in patients with cachexia, when compared to healthy individuals [27]. However, TNF-α’s ability to differentiate between weight losing and weight stable disease in patients is less clear [27]. Similar uncertainty has been demonstrated in studies of oesophageal cancer [25]. The roles of numerous other cytokines, such as interleukin-10, interleukin-1α, interleukin-1β, and interferon-γ have all been similarly examined. Increased expression of many of these has been demonstrated in patients with cancer [28]. It is likely that tumour and host tissues secrete numerous pro-inflammatory mediators that all contribute, to varying levels, to the cachexia process.

### Other mediators of muscle wasting

Activin-A and Myostatin are members of the transforming growth factor beta (TGF-β) family that have been identified as potential tumour-derived catabolic factors in cachexia. Both Activin-A and Myostatin are negative growth factors for skeletal muscle; thus, their overexpression would be likely to contribute towards atrophy. Indeed, higher concentrations of Activin-A, and a positive correlation with weight loss, has been observed in patients with cancer cachexia [33]. While animal models have confirmed that high levels of myostatin are associated with muscle atrophy [34], in studies of humans with cachexia, the results have been more mixed. In a cohort of patients with colorectal or lung cancer, concentrations of myostatin were found to be significantly lower in patients with cancer cachexia compared with weight-stable counterparts [33]. Conversely, in non-weight losing patients with gastric cancer, but not those with lung cancer, expression of myostatin has been found to be significantly higher. This suggests that different tumour types may cause diverse molecular changes in muscle [35].

Macrophage inhibitory cytokine-1/growth differentiation factor 15 (MIC-1 / GDF15) is produced by macrophages in response to their activation and, as such, is expressed at high concentrations during inflammatory states. It has been identified as a hypothalamic modulator of appetite, thus stimulating the loss of both lean and fat mass in animal models [36]. Plasma concentrations of MIC-1/GDF15 have also been noted to be elevated in OG cancer populations [37].

### Hypogonadism

High levels of hypogonadism have been observed in male patients with cancer, especially in those with cachexia [38]. It has been suggested that inflammatory cytokines, and leptin release secondary to fat atrophy, may influence the hypothalamic–pituitary–gonadal axis [39]. Systemic inflammation, measured as CRP and IL-6, and weight loss have been shown to be correlated with hypogonadism in males with unresectable pancreatic cancer [38]. The influence of hypogonadism may account for sexual dimorphism observed in cancer cachexia. Patient sex is known to influence loss of muscle mass, quality and function, with females experiencing attenuated changes in comparison to male counterparts [40]. This may also be a contributing factor towards the high incidence of cachexia in OG cancer, as a male predominant disease. Some evidence has suggested, however, that a proportion of females exhibit hyperoestrogenism and pre-menopausal levels of oestradiol may also be an adverse predictor of survival [38].

### Neuro-endocrine activation

Activation of the neuro-endocrine stress response is well-known to lead to muscle wasting in presence of systemic inflammation [41]. It similarly holds an important role in the physiological response (anorexia and catabolism) to signalling hormones and cytokines seen in cancer cachexia. This is a comparable response to that commonly observed secondary to other forms of illness. Pro-inflammatory stimuli bind to hypothalamic receptors, such as pro-opiomelanocortin and agouti-related protein neurons [14] and stress-responsive adrenal activation causes the release of corticosteroids [41]. Together, these stimulate proteolytic and lipolytic changes across skeletal muscle, cardiac muscle and adipose tissue [42].

### Lipolysis and fat-muscle crosstalk

While earlier cachexia research was predominantly focussed on lean body-mass wasting, more importance is now being placed on changes to visceral and subcutaneous adipose tissue. Increased host energy expenditure necessitates the utilisation of fat stores, via lipolysis, in cancer cachexia [43]. Proinflammatory cytokines and the zinc-α2-glycoprotein (ZAG) lipid-mobilising factor have been explored as potential mediators of this process [44]. Altered gene expression signatures have recently been demonstrated amongst visceral adipose tissue for the adipokines intelectin-1 and intelectin-2, which may be involved in the fat-wasting process in upper GI patients with cancer cachexia [45].

Functional lipolysis may have further relevance to overall cancer cachexia pathways owing to fat-muscle “crosstalk”. Genetic ablation of adipose triglyceride lipase or hormone-sensitive lipase reduced muscle loss in mouse tumour models, suggesting that fat loss is permissive of muscle wasting [46]. This supports the idea that pharmacological inhibition of visceral fat lipolysis may also dampen cachectic changes within skeletal muscle.

### Secondary causes of weight loss

Alongside the primary pathophysiological changes associated with cachexia and anorexia in this OG cancer, there are several secondary factors that may further precipitate wasting. Direct mechanical or digestive issues associated with tumour burden are frequently detrimental to appetite and oral intake. By the time of diagnosis, patients will often have modified their oral intake, to mostly semi-solid food or liquids only, likely further contributing towards poor calorific content.

Neoadjuvant chemotherapy is thought to have a beneficial effect on dysphagia for most patients with oesophageal cancer [47]. The influence of neoadjuvant chemoradiotherapy is more contentious. Complications such as mucositis and oesophagitis, which may worsen dysphagia, can often occur. Secondary analysis of the NeoRes trial showed a decrease in dysphagia scores following treatment with both modalities overall, however noted a significantly higher proportion of patients who had chemoradiotherapy experienced dysphagia following treatment [47]. Despite improved symptoms, weight did not improve in either group. It is possible, therefore, that other effects of neoadjuvant therapy are simultaneously responsible for wasting. Anti-cancer therapies have been shown to disrupt the mTOR kinase pathway, which regulates cell growth and protein anabolism in skeletal muscle [48] and thus may induce muscle wasting. This, followed by the anatomical changes and physiological disruption associated with oesophagectomy or gastrectomy, is likely to compound weight loss in those undergoing potentially curative treatment.

## New paradigms in guiding treatment for oesophagogastric cancer

There is an urgent need to screen for features of the cachectic phenotype in patients with OG cancer. In a potentially curative population, the presence of such features may necessitate modification of treatment pathways or interventions to optimise underlying physiology before and after treatment. For patients with incurable disease, identification and mitigation of the cachexia process could help improve the quality and duration of their remaining life.

Weight loss is one of the most frequently reported presenting symptoms in patients with OG cancer [11, 12]. Pre-treatment weight loss has been shown to have an adverse prognostic impact for patients with gastric or oesophageal cancer [49]. Martin et al. developed a grading system using percentage of weight loss and BMI, which was found to be highly prognostic for survival across a breadth of cancer sites and stages. On subgroup analysis, its discrimination was noted to be particularly effective for gastroesophageal cancers [50]. As such, the presence of weight loss at diagnosis should be considered an important and concerning clinical feature for this patient group.

Sarcopenia is a progressive, generalised disease of skeletal muscle. It is commonly described as an age-related loss of muscle mass and strength, with resultant decline in physical function. However, similar changes can also occur in the context of chronic disease and cachexia. Computed tomography (CT) body composition analysis has allowed researchers to assess patients more readily for sarcopenia. Many consider such objective assessments preferential to anthropometric measures owing to their lack of dependence on patient recall. Recent systematic reviews, considering both curative and palliative cohorts, have identified radiologically-evident sarcopenia as an adverse prognostic marker for survival in both gastric [51] and oesophageal malignancies [52]. The phenomenon of “sarcopenic obesity” has also been of particular interest, with obese patients potentially experiencing progressive sarcopenia that is being overlooked owing to their retained high BMI. Rates of both disease-free and overall survival have been shown to be far worse in this patient group [53]. However, sarcopenia has limitations as a marker of cachexia. A radiological-measurement of sarcopenia on an isolated CT scan is often representative of the patient’s pre-morbid body habitus [54] and does not necessarily reflect dynamic tissue wasting. Low muscle mass and density are commonly present in healthy individuals [55] and endemic throughout a range of cancers and stages [56]. As such, sarcopenia in isolation may be insufficiently prognostic for clinical application. Furthermore, recent analysis of patients with advanced OG cancer, from the EXPAND trial cohort, suggests a lack of causality between sarcopenia and survival [57].

The hepatic acute phase response is stimulated by many of the same inflammatory mediators involved in the pathophysiology of cachexia. As such, laboratory measures of acute phase proteins, such as C-reactive protein (CRP) and albumin, have been used as surrogate indices of inflammation-driven catabolism [14]. In contrast to cytokine assays, these are often routinely measured in clinical practice. The (modified) Glasgow Prognostic Score [58], calculated based on the presence of raised CRP and decreased albumin, has been used extensively to prognosticate in patients with cancer. This has been shown to have particular value in patients with OG malignancies [57, 59]. Alternative scores such as the “prognostic nutritional index”, “platelet: lymphocyte ratio”, and “neutrophil: lymphocyte ratio” have shown similar promise across both operable [60] and inoperable cancers [61].

Despite their efficacy, the utilisation of these biomarkers in current clinical decision-making remains limited. It is likely that phenotypical evidence of cachexia could have value when considering more “borderline” treatment decisions. Further research should aim to explore where best such assessment can effectively guide treatment choices.

## Treat the tumour and treat the host

Despite a growing understanding of cancer cachexia’s pathophysiology, there are a paucity of management strategies in current clinical practice. There is no established standard of care for cachectic patients and, apart from in Japan, there are no licenced drug therapies currently available. All too often, cachexia remains an inevitable and impervious process.

### Influence of cachexia on cancer treatments

A survival benefit is evident for neoadjuvant chemo(radio)therapy in locally advanced OG cancers [3, 4]. These therapies can; however, influence patients’ nutritional and inflammatory state and may theoretically worsen the effects of cachexia. Indeed the CROSS trial, a key study justifying the benefits of neoadjuvant chemoradiotherapy, excluded patients with significant weight loss [3]. CT evaluation has shown both skeletal muscle and fat mass to fall significantly following neoadjuvant therapy for upper gastrointestinal malignancy [62]. Furthermore, there is evidence that the loss of muscle mass during treatment has a greater effect on survival than either pre-treatment or pre-operative sarcopenia in oesophageal [63] and gastric cancer [64]. Decreasing volumes of adipose tissue during neoadjuvant therapy similarly appears to be an adverse marker for survival [65]. Patients with sarcopenia undergoing chemotherapy for oesophageal cancer are also at higher risk for dose-limiting toxicity [66]. Dosing for cytotoxic drugs is commonly calculated based on body surface area, which may not fully reflect a declining lean mass. This discrepancy may result in a decreased volume of distribution and slower drug clearance [67]. Considering these findings, further research is needed to clarify whether cachectic patients get an equivalent benefit from neoadjuvant therapies to weight-stable patients.

The impact of pre-operative cachexia on patients undergoing surgical resection has been evaluated across several observational studies. Chen et al. noted its adverse prognostic impact on survival following gastrectomy, particularly in younger patient groups [68] and similar findings have been noted in patients with early stage oesophageal cancer [69]. Postoperatively, the effects of cachexia can be further compounded by the resultant anatomical changes. Long-term nutritional impairment is particularly prevalent postoperatively, with over one third of patients losing >15% of their body weight in the 5 years following upper gastrointestinal resection [70]. Eating difficulties, dysphagia, nausea, appetite loss, and diarrhoea were all more commonly seen amongst this weight-losing patient group [71], and these chronic symptoms may account for the failure to regain weight.

### Nutritional interventions

Nutritional support is undoubtedly of value in OG cancer care. The symptomatology of the disease puts patients at high risk of poor oral intake even before considering the effects of cachexia. Contemporary guidance stresses the importance of malnutrition screening and dietary counselling in patients with cancer cachexia [72–74]. The use of supplementary enteral nutrition is often necessary in patients with upper GI malignancy. During neo-adjuvant treatment, supported enteral nutrition has shown promise in preventing reduction of muscle mass [75] and postoperative complications [76].

Enteral nutritional support is superior to parenteral, where feasible, following OG resection [72]. In patients who have undergone oesophagectomy, early enteral nutrition reduces the risk of postoperative complications, including anastomotic leak [77]. As such, many centres routinely utilise additional nutritional routes, such as feeding jejunostomy, to provide supplementary postoperative intake. Appetite stimulants, such as corticosteroids and progestins, have been shown to have some short-term efficacy [78]; however, side effects, such as muscle wasting and thromboembolism, limit their clinical use [72]. Especially in more advanced stages of OG cancer, the mechanical components limiting oral intake are compounded by the inflammatory and metabolic consequences of cachexia. While efforts to support nutritional intake are helpful; in isolation they do not sufficiently address the underlying pathophysiology of wasting.

### Medical management of cachexia

Several trials have evaluated treatments targeting mediators of cachexia. Inhibition of the systemic inflammatory response has been a particular area of interest. Pharmacological trials of TNF-α blockade have shown mixed results. Infliximab has not shown benefit, across selected outcomes, for patients with cachexia secondary to pancreatic [79] or lung cancer [80]. Thalidomide, which downregulates TNF-α, alongside cyclo-oxygenase 2, has shown more promise in attenuating weight loss in patients with advanced pancreatic malignancy [81]. While use of an anti-IL-6 monoclonal antibody has shown promise in phase I and II trials for the treatment of cachexia in patients with non-small cell lung cancer [82], further investigation is required to confirm its efficacy. The use of immunonutritional supplements are thought to be beneficial to immune function with the potential to modulate the hyperinflammatory states associated with surgery and cachexia [48]. Omega-3 fatty acids have been trialled following OG cancer resection [83]; however, no effect was observed. Tumour and host tissues likely secrete numerous pro-inflammatory mediators that all contribute, to varying levels, in the cachexia process. As such, trials of unimodal interventions, which target only one of these factors, may not be sufficient to halt systemic inflammation and the resultant wasting.

Both Myostatin and Activin-A share a common receptor: activin-type-2-receptor B. Its antagonism represents another potential therapeutic target for cancer cachexia, with reversed muscle wasting and prolonged survival noted in cancer cachexia murine models treated in such a fashion [84]. Results from human studies trialling an anti-myostatin monoclonal antibody unfortunately did not show similar clinical benefits and, in fact, worse survival was observed in treatment groups [85•]. The efficacy of GDF15 neutralising antibodies is not yet known. A phase 1, first-in-human, trial for patients with advanced stage solid tumours has been completed and recruitment is ongoing for further stages of investigation [86•].

Enobosarm, a non-steroidal selective androgen receptor modulator (SARM), has been evaluated for its anabolic effects on muscle and bone. The POWER II randomised-controlled trial revealed significant improvements in lean body mass, function, and quality of life in older men and post-menopausal women across a number of tumour sites [87]. The relevance of these findings to patients who do not fit this age/sex demographic is unclear at present.

While low-levels of the appetite-stimulating hormone ghrelin do not appear to be associated with cancer cachexia in human studies [88], ghrelin agonists have been trialled in the treatment of cachexia. Anamorelin, a ghrelin agonist, is now licenced for use in Japan for patients with cancer cachexia secondary to a number of tumour types, including gastric [73]. The ROMANA 1&2 trials identified an increase in lean body mass and an improved symptom burden in patients treated using this drug [89, 90••]. However, grip strength, which was also selected as an endpoint for this trial, was not affected. It could be suggested that heterogeneity of chosen outcome measures may be limiting progress in cachexia trials. It is imperative that consensus is reached on appropriate, clinically relevant endpoints.

Overall, medical therapies for cachexia have shown modest efficacy in human studies. High-quality randomised-controlled-trials are still required to identify effective disease-modifying therapies.

### Exercise-based therapies

Physical exercise has the potential to improve muscle mass and function in patients with cancer cachexia; however, robust evidence is currently lacking [91], particularly in patients with advanced stage malignancy [92]. Trials have high rates of attrition, often owing to disease progression but interventions appear to be safe when appropriately supervised [73]. At the time of writing, the full results from the “ChemoFit” prehabilitation study are still awaited [93]; however, early findings suggest that exercise-interventions alone may not be preventative for sarcopenia during neoadjuvant chemotherapy for OG cancer [94].

### Multimodal interventions

Trials of nutritional and exercise-based interventions, in isolation, for patients undergoing resection of upper GI cancer have yielded inconsistent results [95, 96]. As Fearon suggested over a decade ago; cachexia is a multi-factorial syndrome that likely requires a multimodal intervention [97]. Alongside anti-cancer therapies, supportive management requires a personalised approach with psychological and social support, nutritional, physical, anti-catabolic, and anti-inflammatory treatments. Although currently only recruiting in lung and pancreatic cancer, there is hope that multimodal trials such as MENAC (Multimodal—Exercise, Nutrition and Anti-inflammatory medication for Cachexia) will return promising results [98•]. Combining resistance and aerobic training, dietary counselling, oral supplements, and suppression of the inflammatory process may provide more cumulative efficacy in preventing, mediating, or even reversing the effects of cancer cachexia. Such interventions could yield benefit throughout the disease trajectory of OG cancer.
